# Supplementary material for: Prevalence and determinants of inadequately controlled diabetes in Qatar’s public primary care settings: A cross-sectional study
Source: PLoS One. 2026 Apr 9;21(4):e0346266. doi: 10.1371/journal.pone.0346266 (PMC13065076; doi:10.1371/journal.pone.0346266)
Supplement: Attachment — Submitted filename: REVIEWERS PLOS One Qatar 2.docx [file pone.0346266.s001.docx]

REVIEWER’S COMMENTS**: Prevalence and determinants of inadequately controlled diabetes in Qatar’s public primary care settings: A five-year cross-sectional review of health**

**records (2018-2022)**

Dear Editor,

Thank you for inviting me to reviewer manuscript by Sameer *et al* on Prevalence and determinants of inadequately controlled diabetes in Qatar’s public primary care settings: A five-year cross-sectional review of health records (2018-2022).

**Title and Abstract**

**Title**: Clear and concise

**Abstract:** The abstract summarizes the study well, highlighting research problem and gaps clearly.

However, background should be summarized further and conclusion should be revise to include policy implication of findings, while deleting the recommendation as subheading.

**Introduction**: Well written and conceptualizes the research problem effectively. Nonetheless, there is need for revision to include literature review on prevalence of uncontrolled T2DM in middle east and other parts of the world.

**Methodology**: Methodology is generally sound and this will allow repeatability of the study and readers to quickly understand the study protocol. This helps establish validity and reliability of findings.

**Results**: The results are well presented and supported by appropriate statistical analysis. Moreover, there is need to revise first paragraph where the sentence starts with **Of the eligible**….. to maintain academic tone. HbA1C>=7% should be replace with standard abbreviation of $\geq.$

Tables and figures: Tables are well organized and figure caption described the information clearly however, correct the HbA1C>=7% to standard abbreviation of HbA1C $\geq7\%.$

**Discussion**: The discussion is well structured and effectively compares the findings with previous research, highlighting limitations and recommendations.

**Conclusion**: The conclusion summarizes the study results effectively, including policy implication of the study.

**References:** The references are appropriate and relevant \, Moreover, ensure compliance with the journal guidelines.

**Remarks**: The study will contribute valuable scientific data considering a robust sample size from population-based study. Findings from this study may guide policy on relevant interventions to address the burden of uncontrolled DM and minimized complications.
