# Supplementary material for: Prevalence and determinants of inadequately controlled diabetes in Qatar’s public primary care settings: A cross-sectional study
Source: PLoS One. 2026 Apr 9;21(4):e0346266. doi: 10.1371/journal.pone.0346266 (PMC13065076; doi:10.1371/journal.pone.0346266)
Supplement: Attachment — Submitted filename: Response to Reviewers comments-R2.docx [file pone.0346266.s005.docx]

Reviewer #3: The authors did not respond to many of the further below reviewer comments. Defer data availability appropriateness to editorial team.

--line 97: is there statistical rationale for this comorbidity score approach?

**Author’s response**: The comorbidity score is a non-biased statistical approach for studying the burden of comorbid conditions in a diabetic individual. The selected comorbidities are known to occupy the highest rank in a long list of comorbid conditions associated with DM. In addition, the selected conditions can be easily and accurately captured as diagnostic codes by the managing physician in the electronic health record system of the PHCC.

--line 107: acknowledge use of <7% as general A1c goal, but <7.5 or <8% can often be used as a goal in geriatric populations. 33% of this cohort was greater than or equal to 70 years old. can you complete any analyses in this demographic for goal A1c <7.5% or <8%? Fine for supplemental here..

**Author’s response**: This is an interesting comment. Relaxing the HbA1c cut-off value for proper DM control to 7.5% or 8% is decided on a case-by-case basis for those older than 70 years depending on their overall health status. This is not an absolute requirement, but realistic accommodation for the compromised capacity of older age group. Including such an adjustment in calculating the population level prevalence of inadequately controlled diabetes would introduce bias and limit the comparison with other studies. In addition, since the focus of this study is to portray the primary care system performance in managing DM it would be more conservative to use the global HbA1c criteria of <7%.

To satisfy our scientific curiosity the table below will demonstrate the trivial impact of adjusting the cut-off value for older ages. For example, increasing the cut-off value of HbA1c for defining inadequately controlled DM in older ages (70+ years) to 7.5% would reduce the overall prevalence rate by 2% in the year 2018 compared to the global estimate. In addition, further increasing the cut-off value to 8% would reduce the prevalence rate by 4% in the year 2018.

| **Prevalence rate of Inadequately controlled DM by year** | **2018** | **2019** | **2020** | **2021** | **2022** |
| --- | --- | --- | --- | --- | --- |
| Global criteria (HbA1C>=7%) | 58.8 | 55.3 | 53.2 | 53.5 | 53.9 |
| Adjusted for older age (70+ years) exception (HbA1C>=7%, except for ages 70+ [>=7.5%]) | 56.5 | 53.1 | 51.3 | 51.9 | 52.4 |
| Adjusted for older age (70+ years) exception (HbA1C>=7%, except for ages 70+ [>=8%]) | 54.5 | 51.4 | 49.9 | 50.6 | 51.2 |

--line 122: capitalization?

**Author’s response**: Done.

--line 296: T2DM

**Author’s response**: Done.

--line 297: no need to capitalize calendar

**Author’s response**: Done.

From prior (with updated line numbers):

General:

--Were individuals with anemia excluded since anemia can cause a falsely high A1c?

**Author’s response**: No exclusion of anemia was practiced. We will report this as a study limitation.

--Were pregnant individuals excluded since A1c levels generally decrease during pregnancy?

**Author’s response**: Pregnant females were excluded. We will update this in the study population.

--any review of pharmacotherapy or diabetes incidence? if not, why not?

**Author’s response**: I’m sorry we did not understand the comment correctly. Why do we need a review of pharmacotherapy or diabetes incidence to describe the epidemiology of inadequately controlled DM. The focus of the manuscript is the health system and primary care in specific and not the type of treatment and its performance. Nevertheless, It’s a good suggestion for a new research idea about DM.

--abstract and throughout (lines 180-185): While acknowledged in lines 285-290, inadequately controlled T2D is a clinical risk factor or predictor of retinopathy (and chronic kidney disease and coronary disease), not vice versa.

**Author’s response**: The term “RISK” is a statistical term referring to the strength of association between an explanatory and an outcome variables. It’s written as risk but refers to probability. This study is cross-sectional which fails to show a directional relationship between the variables. That is why statistical risk is a measure of effect size and is different from clinical risk in which the cause precedes the effect. However, the term risk was changed to probability to reduce the confusion.

--How does the study extend: <https://www.gavinpublishers.com/article/view/factors-associated-with-glycemic-control-among-type-2-diabetic-patients-attending-primary-health-care-centers-in-qatar-a-cross-sectional-study> ?

**Author’s response**: The study referred to can be considered as a small pilot project with convenient sample that highlighted the importance of uncovering the reasons behind having a substantial size of uncontrolled DM. The current study included a huge study population, which can describe the inadequately controlled DM at the wider primary health care system.

Lined:

--line 63: prefer adults with diabetes

**Author’s response**: Done.

--line 155: (Table 1)

**Author’s response**: Done.

--line 109: What is the rationale for "mean annual A1c" instead of categorizing as possible into newly controlled, newly uncontrolled, or persistently uncontrolled, or persistently controlled cohorts?

**Author’s response**: It’s a known statistical assumption that mean is the most unbiased representation of a sample of quantities that are known or assumed to be normally distributed. Since HbA1c is a cumulative representation of blood glucose throughout a period of at least 3 months, it is unlikely for the two or probably measurements taken during a year to show extreme fluctuations that may bias the calculation of the mean. The mean HbA1c qualifies as a good index representing diabetes control over a year. The suggested terminologies of newly controlled, newly uncontrolled, or persistently uncontrolled, or persistently controlled cohorts may look plausible, but are difficult to defend and justify when a specific cut-off value is addressed.

--lines 252-258: unclear with the distinctions here that seem to require dedicated statistical review of the models as they contribute to confusion throughout (e.g., lines 173-175 and Table 1 describe risk reduction but Table 2 shows increased ORs of inadequately controlled A1c; "Aging increases the risk of having inadequately controlled T2DM, but this was attributed to comorbidities. Adjusting for those comorbidities will show that younger ages are the ones at higher risk.")

**Author’s response**: We have consulted a biostatistician to review the results of multivariate models before releasing the manuscript for a scientific publication. Can you please clarify what is needed from our end?
