# Supplementary material for: Prevalence and determinants of inadequately controlled diabetes in Qatar’s public primary care settings: A cross-sectional study
Source: PLoS One. 2026 Apr 9;21(4):e0346266. doi: 10.1371/journal.pone.0346266 (PMC13065076; doi:10.1371/journal.pone.0346266)
Supplement: Attachment — Submitted filename: Response to reviewers.docx [file pone.0346266.s006.docx]

PONE-D-25-42745R2

Prevalence and determinants of inadequately controlled diabetes in Qatar’s public primary care settings: A cross-sectional study

**Reviewer #3: 6. Review Comments to the Author**

**Reviewer #3:** Appreciate the authors' thoughtful responses that clarify. Some minor comments as below.

-Would suggest mentioning negligible effects in analyses of higher geriatric A1c goals in the discussion

**Author’s response**: The below paragraph was added to the first part of discussion to address the reviewer’s comment:

One final consideration in this context is the American Diabetes Association’s recommendation to relax HbA1c treatment targets in older adults, allowing goals of 7.5% or even 8% to account for age-related limitations. These adjustments are made on a case-by-case basis. In this study, prevalence estimates were calculated using the universally accepted HbA1c threshold of 7% to ensure comparability with other published reports. However, under the extreme hypothetical assumption that all individuals aged 70 years and above were assessed using an 8% threshold, the prevalence of inadequately controlled diabetes in 2018 would decline by only 4%[24].

-Pharmacotherapy and T2D incidence (and newly controlled, newly uncontrolled, or persistently uncontrolled, or persistently controlled cohorts) were suggested to clarify as able if "inadequately controlled T2D" was related to newly diagnosed uncontrolled diabetes that subsequently became controlled rather than chronically uncontrolled diabetes. Fine not to include per authors' response; fine to include in limitations or future directions if authors think helpful.

**Authors response:** This new paragraph was added to the “Recommendations section”:

Lastly, future research should address the confounding influence of pharmacotherapy and the categorization of T2DM patients into cohorts defined as newly controlled, newly uncontrolled, persistently uncontrolled, or persistently controlled.

-For participants with three or more A1c values in a year, were A1c values normally distributed in each of the five years? This seems that it would most best justify the role of the mean annual A1c ("three or more values" to allow a limited within-participant normality test)

**Authors response:** Thank you for posting this comment. Let me refer to a known statistical theory called the “central limit theorem”. It states that the mean will always be as close as possible to a normal distribution for very large samples, which is the case in this study.

-line 66: six

**Authors response:** Done

-line 96 and Table 1: "comorbidity score" description could be deleted and Table 1 just state "number of comorbidities" to more directly describe approach but fine to defer to authors' preferences

**Authors response:** The new version of the statement is as follows:

Summing positive comorbidities per individual created a new variable (number of comorbidities) to reflect the health burden.

The “comorbidity score” (in table 1) was changed to “number of comorbidities”.

-line 147: why highlighted?

**Authors response:** Honest oversight.

-line 156, 298: T2DM

**Authors response:** Done
